# Baseline Morbidity in 2,990 Adult African Volunteers Recruited to Characterize Laboratory Reference Intervals for Future HIV Vaccine Clinical Trials

**DOI:** 10.1371/journal.pone.0002043

**Published:** 2008-04-30

**Authors:** Wendy Stevens, Anatoli Kamali, Etienne Karita, Omu Anzala, Eduard J. Sanders, Walter Jaoko, Pontiano Kaleebu, Joseph Mulenga, Len Dally, Pat Fast, Jill Gilmour, Bashir Farah, Josephine Birungi, Peter Hughes, Olivier Manigart, Gwynn Stevens, Sarah Yates, Helen Thomson, Andrea von Lieven, Marietta Krebs, Matt A. Price, Lisa Stoll-Johnson, Nzeera Ketter

**Affiliations:** 1 University of Witwatersrand and Contract Laboratory Services (CLS), Johannesburg, South Africa; 2 Medical Research Council (MRC), Uganda Virus Research Institute (UVRI) Unit on AIDS, Entebbe and Masaka, Uganda; 3 Projet San Francisco (PSF), Kigali, Rwanda; 4 Kenya AIDS Vaccine Initiative (KAVI), Kenyatta National Hospital and Kangemi, Nairobi, Kenya; 5 Centre for Geographic Medicine Research-Coast (CGMRC), Kenya Medical Research Institute (KEMRI), Kilifi, Kenya; 6 Zambia Emory HIV Research Project (ZEHRP), Lusaka, Zambia; 7 The EMMES Corporation, Washington, D.C. United States of America; 8 International AIDS Vaccine Initiative (IAVI), New York, New York, United States of America; 9 Uganda Virus Research Institute (UVRI) / International AIDS Vaccine Initiative (IAVI) HIV Vaccine Program, Entebbe, Uganda; 10 Novartis, Boston, Massachusetts, United States of America; 11 Johnson and Johnson, New Brunswick, New Jersey, United States of America; University of Liverpool, United Kingdom

## Abstract

**Background:**

An understanding of the health of potential volunteers in Africa is essential for the safe and efficient conduct of clinical trials, particularly for trials of preventive technologies such as vaccines that enroll healthy individuals. Clinical safety laboratory values used for screening, enrolment and follow-up of African clinical trial volunteers have largely been based on values derived from industrialized countries in Europe and North America. This report describes baseline morbidity during recruitment for a multi-center, African laboratory reference intervals study.

**Methods:**

Asymptomatic persons, aged 18–60 years, were invited to participate in a cross-sectional study at seven sites (Kigali, Rwanda; Masaka and Entebbe, Uganda; Kangemi, Kenyatta National Hospital and Kilifi, Kenya; and Lusaka, Zambia). Gender equivalency was by design. Individuals who were acutely ill, pregnant, menstruating, or had significant clinical findings were not enrolled. Each volunteer provided blood for hematology, immunology, and biochemistry parameters and urine for urinalysis. Enrolled volunteers were excluded if found to be positive for HIV, syphilis or Hepatitis B and C. Laboratory assays were conducted under Good Clinical Laboratory Practices (GCLP).

**Results and Conclusions:**

Of the 2990 volunteers who were screened, 2387 (80%) were enrolled, and 2107 (71%) were included in the analysis (52% men, 48% women). Major reasons for screening out volunteers included abnormal findings on physical examination (228/603, 38%), significant medical history (76, 13%) and inability to complete the informed consent process (73, 13%). Once enrolled, principle reasons for exclusion from analysis included detection of Hepatitis B surface antigen (106/280, 38%) and antibodies against Hepatitis C (95, 34%). This is the first large scale, multi-site study conducted to the standards of GCLP to describe African laboratory reference intervals applicable to potential volunteers in clinical trials. Approximately one-third of all potential volunteers screened were not eligible for analysis; the majority were excluded for medical reasons.

## Introduction

Africa has the largest burden of HIV infection and AIDS worldwide [1]. Laboratory reference intervals for healthy populations have not been formally established in most African countries and consequently “Western” laboratory reference intervals, derived from predominantly Caucasian populations in Western Europe and the United States, are most often used to determine whether individual laboratory values should be defined as normal or out-of-range. Consequently, significant numbers of potential volunteers are often excluded. Therefore, it is important to better define the ranges of laboratory values found in healthy adults likely to enroll in future trials [2], [3]. There is some evidence from small studies conducted in eastern, southern and northern African populations that differences do exist between “Western” reference intervals and those of adult Africans considered to be healthy [4]–[7]. In many studies however, health status was usually determined by interview alone and did not include physical examination or extensive medical history, pregnancy testing or evaluation for certain clinical conditions.

In order to prepare a vaccine trial laboratory network following Good Clinical Laboratory Practices (GCLP) and to define a set of reference intervals suitable for future trial participants, the International AIDS Vaccine Initiative, the Medical Research Council in Uganda, the Rwanda-Zambia HIV Research Group, the Kenyan AIDS Vaccine Initiative and the Kenyan Medical Research Institute initiated a cross-sectional survey. The laboratory approach to this study was modeled on the Clinical and Laboratory Standards Institute (CLSI) guidelines for determining reference values and intervals for quantitative clinical laboratory tests [8].

This paper presents the recruitment, enrollment, and baseline morbidities of the 2,990 potential study volunteers.

## Methods

### Study volunteers

**Figure 1:**
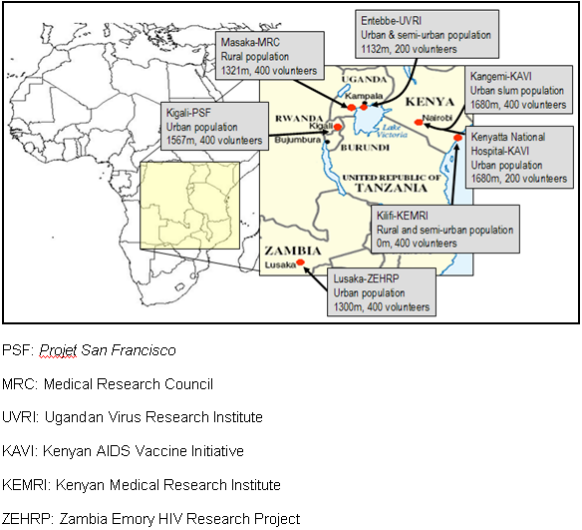
Map of study sites showing source populations, elevation (meters above sea level) and target enrolment.

Clinically healthy adult (18–60 years) male and female volunteers were enrolled across seven sites in four countries in Eastern and Southern Africa (Figure 1). All potential volunteers had received HIV Voluntary Counseling and Testing (VCT) and had a negative HIV test within three months prior to screening for this study. Eligibility criteria for this study were similar to those used for HIV vaccine clinical trials and source populations were selected as described below. Target enrollments for all sites were 200 or 400 volunteers, depending on site capacity, with equal numbers of men and women.

#### Masaka-Medical Research Council (MRC)/ Ugandan Virus Research Institute (UVRI) Unit on AIDS, Uganda

Eligible volunteers were selected from a rural general population cohort enrolled into prospective HIV incidence studies in preparation for HIV vaccine trials.

#### Entebbe-UVRI, Uganda

Volunteers for this study were drawn from community members who: 1) had expressed interest to participate in future clinical trials, or 2) were prescreened for a previous HIV vaccine phase 1 clinical trial and were not enrolled because the trial had completed enrollment.

#### Kilifi-Kenya Medical Research Institute (KEMRI), Kenya

Half of this site's study volunteers were drawn from an HIV prevalence study in Kilifi Town, and half were selected from individuals who were enrolled in HIV incidence studies in preparation for HIV vaccine trials.

#### Kangemi-Kenya AIDS Vaccine Initiative (KAVI), Kenya

Volunteers were drawn from an HIV prevalence study conducted in this peri-urban district of Nairobi in preparation for HIV incidence studies.

#### Kenyatta National Hospital (KNH)-KAVI, Kenya

The majority of volunteers from this site included medical students, staff and professionals from the KNH medical school and hospital facility. Community members not affiliated with the facility were also enrolled.

#### Lusaka-Zambia Emory HIV Research Program (ZEHRP), Zambia and Kigali-*Projet San Francisco* (PSF), Rwanda

Half of the volunteers from these two sites were drawn from large prospective studies of long-term, stable sexually active couples of HIV discordant status (the volunteer's partner was HIV positive), and half were drawn from couples identified during couples' VCT as concordant HIV-negative (both partners HIV uninfected).

### Study procedures

This study was approved by the Institutional Ethics Committees (EC) or Institutional Review Boards (IRB) at each participating institution, including Emory University; all institutions have an EC/IRB that is registered with the US Office of Human Research Protection.

Interested potential volunteers were administered a brief screening questionnaire and symptom-directed examination prior to enrollment. Volunteers were screened out based on significant medical history including current clinical symptoms, immunosuppressive or corticosteroid medication, chemotherapy, hospitalizations, surgery or blood transfusions in the six months prior to screening. Volunteers with splenomegaly (Grade 2+ by Hackett's classification) were excluded. Menstruating women were asked to return in two weeks, and women who reported being pregnant were not enrolled. Breastfeeding was not an exclusion factor. No personal identifying information was collected from volunteers who were screened out prior to enrollment; only age, gender and reason for ineligibility.

Following screening, written informed consent was obtained from all eligible volunteers. The consent process included an explanation and discussion of the study procedures, followed by an assessment of the potential volunteer's understanding of the study. Literacy was not a requirement to participate, and illiterate volunteers were consented with an independent third party present to confirm volunteer understanding of the consent process and study procedures. Only those volunteers who could demonstrate a satisfactory understanding following the consenting process were enrolled.

After enrollment, a detailed medical history including reproductive history for women, data on contraception use, investigation of current medications and demographics (socioeconomic status, education, environmental exposures, smoking, and drug and alcohol consumption) were collected from each enrolled volunteer. A physical examination was performed including evaluation of vital signs, weight and height. Blood was drawn for HIV, syphilis and Hepatitis C serology, Hepatitis B antigen, hematology (complete blood count), clinical chemistry (aspartate aminotransferase, alanine aminotransferase, total and direct bilirubin, albumin, total immunoglobulin, creatinine, amylase, creatinine phosphokinase, lactate dehydrogenase, alkaline phosphatase and total protein), and CD4/CD8 T-cell count. The assays used are outlined in Table 1. The HIV testing algorithm at most sites used two concurrent rapid HIV tests followed by a confirmatory ELISA if either rapid test was positive. Urinalysis was performed, and urine pregnancy tests performed in women. If needed treatment was not available on-site, volunteers were referred for appropriate care. Enrolled volunteers were excluded from subsequent analysis if the laboratory tests revealed that they were pregnant, positive for HIV-1 or HIV-2, Hepatitis B surface antigen (HBsAg), antibodies against hepatitis C or RPR (suspected syphilis).

**Table 1:** Laboratory assays used for screening enrolled volunteers.

Site	Hepatitis B	Hepatitis C	HIV	Pregnancy	Syphilis
Kigali-PSF	HBsAG ELISA (Abbot-Murex version 3)	Anti-HCV (Abbot-Murex version 4)	Rapid HIV 1/2 Determine (Abbott), Rapid HIV 1/2 Capillus (Trinity Biotech), HIV 1/2 ELISA Vironostika Uni-Form II Ag/Ab (Biomerieux)	ßhCG reagent strips (Bayer Multistix 10SG), Cypress-hCG Dipstrip	RPR Carbon (Spinreact)
Masaka-MRC	Hepanostika HBsAg Uni-Form II MicroELISA system (Biomerieux)	Innotest HCV Ab IV (Innogenetics)	Rapid HIV 1/2 Determine (Abbott), HIV 1/2 ELISA Vironostika Uni-Form II Ag/Ab (Biomerieux), Murex HIV-1.2.0 ELISA (Abbott), HIV-1 Western Blot Kit (Calypte biomedical)	ßhCG reagent strips (Bayer Multistix 10SG), Hexagon hCG 1-Step, Cypress Diagnostics hCG slide	RPR Test (Biotec)
Kilifi-KEMRI	Hepanostika HBsAg Uni-Form II MicroELISA system (Biomerieux)	Innotest HCV Ab IV (Innogenetics)	Rapid HIV 1/2 Determine (Abbott), Rapid HIV 1/2 Uni-Gold (Trinity Biotech), discrepant results sent for confirmation at KNH-KAVI	ßhCG reagent strips (Bayer Multistix 10SG)	Macro-Vue RPR Test (Becton Dickson) with TPHA confirmation
Kangemi-KAVI	Hepanostika HBsAg Uni-Form II MicroELISA system (Biomerieux)	Innotest HCV Ab IV (Innogenetics)	Rapid HIV 1/2 Determine (Abbott), Rapid HIV 1/2 Uni-Gold (Trinity Biotech), discrepant results sent for confirmation at KNH-KAVI	ßhCG reagent strips (Bayer Multistix 10SG), Hexagon hCG 1-Step	RPR Test (Forest Diagnostics Ltd)
Kenyatta National Hospital-KAVI	Hepanostika HBsAg Uni-Form II MicroELISA system (Biomerieux)	Innotest HCV Ab IV (Innogenetics)	Rapid HIV 1/2 Determine (Abbott), Rapid HIV 1/2 Uni-Gold (Trinity Biotech), HIV 1/2 ELISA Vironostika Uni-Form II Ag/Ab (Biomerieux), Detect-HIV ELISA (Adaltis, Inc)	ßhCG reagent strips (Bayer Multistix 10SG), Hexagon hCG 1-Step	RPR Test (Forest Diagnostics Ltd)
Entebbe-UVRI	Hepanostika HBsAg Uni-Form II MicroELISA system (Biomerieux)	Innotest HCV Ab IV (Innogenetics)	Rapid HIV 1/2 Determine (Abbott), HIV 1/2 ELISA Vironostika Uni-Form II Ag/Ab (Biomerieux), Murex HIV-1.2.0 ELISA (Abbott), Cambridge Biotech HIV-1 Western Blot Kit (Calypte biomedical),	Hexagon hCG 1-Step	RPR Test (Biotec)
Lusaka-ZEHRP	HBsAG ELISA (Abbot-Murex version 3)	Anti-HCV (Abbot-Murex version 4)	Rapid HIV 1/2 Determine (Abbott), Rapid HIV 1/2 Capillus (Trinity Biotech), Murex HIV-1.2.0 ELISA (Abbott), HIV 1/2 ELISA Vironostika Uni-Form II Ag/Ab (Biomerieux)	ßhCG reagent strips (Bayer Multistix 10SG), Hexagon hCG 1-Step	RPR Antigen Suspension (Becton Dickson)

### Clinical Laboratories and Assays

For comparative evaluation across centers, standardization of methods and equipment was deemed essential, and equipment was chosen that is easy to operate, suitable for lower-volume work and low-maintenance. For hematology measurements, all sites used the Beckman Coulter Act5 Diff analyzer (Beckman Coulter, USA). Biochemistry assays were performed on the VitaLab Selectra E (VitalScientific, The Netherlands). Both instruments had suppliers within the region to ensure regular service, maintenance and reagent supply. The two KAVI sites shared the same laboratory facilities at the KNH. Five laboratories used the FACSCount (BD Biosciences) due to its robust performance on the WHO/ NHLS CDREQAS program across African sites [9]; the KAVI laboratory used the FACSCalibur flow cytometry system (BD Biosciences) for CD4 and CD8 counts. To minimize any contribution of diurnal variation [10], samples for CD4 counts were taken before noon. The study was conducted according to principles of GCLP [11] to ensure data reproducibility and reliability and to prepare site laboratories for the requirements of trial regulatory bodies.

### Quality Assurance and Quality Control

Staff received GCLP training as well as specific technical training on each analyzer depending on individual site requirements [8], [11]. All laboratory procedures were formalized in standard operating procedures. Site audits were conducted prior to study initiation and during the course of the study to ensure that pre-defined GCLP standards were maintained. This approach to establishing laboratories has been described previously by Gilmour and colleagues [12].

A central reference laboratory in Johannesburg, Republic of South Africa, assisted with the conduct of cross-validation studies which included the selection and shipment of proficiency panels of 60 samples from the reference centre to the sites for biochemistry, hematology and urinanalysis. Results were compared with the reference laboratory, across technicians, and across sites using the Bland-Altman [13] and the Percentage Similarity methods [14]. All sites were enrolled on External Quality Assurance (EQA) programs provided by the National Health Laboratory Service in South Africa for hematology, chemistry, serology and CD4 counts.

### Data

Data were transcribed onto case report forms (CRF) scanned and emailed to a central server using DataFax (Clinical DataFax Systems Inc., Hamilton, Canada). Quality assurance included on-site monitoring of source documents and CRFs, and automated checks of the electronic data. Data analyses were conducted using Stata (College Park, TX, USA) and SAS (Cary, NC, USA) software. Results are descriptive and include tabulations of screened and enrolled volunteers, and baseline study population characteristics. Appropriate statistical tests (Wilcoxon rank sum, Fisher's exact test) are shown by their p-value.

## Results

**Figure 2:**
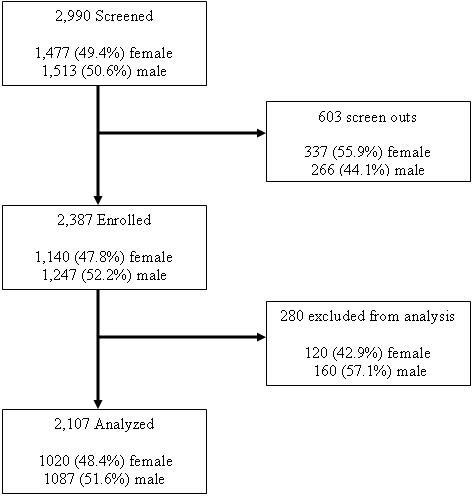
Equivalent numbers of men and women aged 18-60 were screened for this study. Among the 2,990 volunteers screened, 2,387 were enrolled, and a further 263 were excluded from analysis leaving a final study cohort of 2,124 volunteers.

Screening and enrollment began in December 2004 and ended in October 2006. A total of 2990 individuals were screened across all sites, 1477 women (49.4%) and 1513 men (50.6%). Approximately 20% of screened volunteers were not enrolled with a further 10% excluded following enrollment (Figure 2). There was considerable variability in the screen-out and exclusion rates across sites (Table 2). More women were screened out than men (22.8% versus 17.6%, Fisher's exact 2-tailed test: p<0.001), and this was consistent (though not always statistically significant) across all sites except Entebbe. Volunteers who were screened out tended to be older than enrolled volunteers (median age: 30 vs. 28 years, Wilcoxon 2-sample test: p = 0.001).

**Table 2:** Screened, enrolled and analyzed volunteers by site and gender.

	Kigali-PSF		Kangemi-KAVI		Kenyatta National Hospital -KAVI		Entebbe-UVRI		Masaka-MRC		Lusaka-ZEHRP		Kilifi-KEMRI		Study Totals	
	N	%	N	%	N	%	N	%	N	%	N	%	N	%	N	%
**Total**																
Screened	505	–	434	–	214	–	230	–	602	–	497	–	508	–	2990	–
Enrolled	400	79.2%	396	91.2%	204	95.3%	222	96.5%	405	67.3%	393	79.2%	367	72.2%	2387	79.8%
Analyzed	373	73.9%	362	83.4%	197	92.1%	194	84.3%	333	55.3%	352	70.8%	296	58.3%	2107	70.5%
**Female**																
Screened	263	–	215	–	111	–	110	–	293	–	252	–	233	–	1477	–
Enrolled	198	75.3%	193	89.8%	104	93.7%	108	98.2%	183	62.5%	197	78.2%	157	67.4%	1140	77.2%
Analyzed	188	71.5%	176	81.9%	99	89.2%	98	89.1%	146	49.8%	184	75.1%	129	55.4%	1020	69.9%
**Male**																
Screened	242	–	219	–	103	–	120	–	309	–	245	–	275	–	1513	–
Enrolled	202	83.5%	203	92.7%	100	97.1%	114	95.0%	222	71.8%	196	80.3%	210	76.4%	1247	82.4%
Analyzed	185	76.4%	186	84.9%	98	95.1%	96	80.0%	187	60.5%	168	68.6%	167	60.7%	1087	72.2%

The most common reasons for screen-outs prior to enrollment were splenomegaly (89/603, 14.8%), inability to demonstrate satisfactory comprehension during the informed consent process (75, 12.4%), hypertension (61, 10.1%), symptoms of upper respiratory infection (51, 8.5%) and menstruating women who did not return for re-screening (44, 7.3%). Some volunteers had more than one reason for exclusion. Most potential volunteers had been pre-screened for HIV; only 3 potential volunteers were found to be HIV infected at screening. Table 3 shows the prevalence of each exclusionary criterion as a proportion of all volunteers screened. Once enrolled, slightly more men than women were excluded from analysis (12.8% versus 10.5%, Fisher's exact 2-tailed test: p = 0.06), due to a higher prevalence of Hepatitis B surface antigen (5.5% vs 3.1%, p = 0.002) and Hepatitis C antibody (6.7% vs. 4.2%, p = 0.005) in men. The final sample of 2107 volunteers was 48.4% women, 51.6% men (Table 2).

**Table 3:** Summary of reasons for screen-outs and exclusion from analysis by site.

	Kigali- PSF		Kangemi-KAVI		Kenyatta National Hospital-KAVI		Entebbe-UVRI		Masaka- MRC		Lusaka-ZEHRP		Kilifi- KEMRI		Study Totals	
	N	%	N	%	N	%	N	%	N	%	N	%	N	%	N	%
**Total number of volunteers screened**	**505**		**434**		**214**		**230**		**602**		**497**		**508**		**2990**	
**Volunteers screened out ** *	**105**	**20.8%**	**38**	**8.8%**	**10**	**4.7%**	**8**	**3.5%**	**197**	**32.7%**	**104**	**20.9%**	**141**	**27.8%**	**603**	**20.2%**
Splenomegaly	7	1.4%	0	0.0%	0	0.0%	2	0.9%	78	12.9%	1	0.2%	1	0.2%	89	3.0%
Hypertension**	6	1.2%	9	2.1%	0	0.0%	0	0.0%	22	3.6%	22	4.4%	2	0.4%	61	2.0%
Flu like symptoms ***	9	1.8%	6	1.4%	0	0.0%	0	0.0%	22	3.6%	7	1.4%	7	1.4%	51	1.7%
Sexually transmitted infection ****	4	0.8%	0	0.0%	1	0.5%	0	0.0%	21	3.5%	9	1.8%	2	0.4%	37	1.2%
Low body-mass index	13	2.6%	0	0.0%	0	0.0%	1	0.4%	11	1.8%	7	1.4%	1	0.2%	33	1.1%
Acute respiratory infections ‡	8	1.6%	7	1.6%	1	0.5%	1	0.4%	7	1.2%	3	0.6%	5	1.0%	32	1.1%
HIV antibody positive	1	0.2%	0	0.0%	0	0.0%	0	0.0%	0	0.0%	1	0.2%	1	0.2%	3	0.1%
Other medical history/exam reasons †	32	6.3%	17	3.9%	6	2.8%	6	2.6%	45	7.5%	39	7.8%	39	7.7%	184	6.2%
Menstruating, did not return	13	2.6%	6	1.4%	1	0.5%	0	0.0%	4	0.7%	18	3.6%	2	0.4%	44	1.5%
Pregnant	0	0.0%	1	0.2%	1	0.5%	0	0.0%	11	1.8%	2	0.4%	12	2.4%	27	0.9%
Unable to complete informed consent	17	3.4%	3	0.7%	0	0.0%	0	0.0%	27	4.5%	2	0.4%	26	5.1%	75	2.5%
Other non-medical reasons	8	1.6%	1	0.2%	0	0.0%	0	0.0%	8	1.3%	8	1.6%	56	11.0%	81	2.7%
**Total number of enrolled volunteers**	**400**		**396**		**204**		**222**		**405**		**393**		**367**		**2387**	
**Volunteers excluded after enrollment** *	**27**	**6.8%**	**34**	**8.6%**	**7**	**3.4%**	**28**	**12.6%**	**72**	**17.8%**	**41**	**10.4%**	**71**	**19.3%**	**263**	**11.0%**
Hepatitis B antigen positive	13	3.3%	13	3.3%	4	2.0%	10	4.5%	5	1.2%	23	5.9%	38	10.4%	106	4.4%
Hepatitis C antibody positive	10	2.5%	4	1.0%	0	0.0%	10	4.5%	37	9.1%	6	1.5%	28	7.6%	95	4.0%
Syphilis / RPR positive	0	0.0%	12	3.0%	0	0.0%	8	3.6%	21	5.2%	11	2.8%	3	0.8%	55	2.3%
Pregnant	4	1.0%	5	1.3%	1	0.5%	0	0.0%	2	0.5%	1	0.3%	5	1.4%	18	0.8%
Other §	0	0.0%	3	0.8%	1	0.5%	0	0.0%	11	2.7%	2	0.5%	0	0.0%	17	0.7%

Percentages shown as a proportion of either total screened (above), or total enrolled (below)

* Volunteers may be excluded for multiple reasons therefore columns may sum to >100%

** Systolic blood pressure >140 mm Hg and/or diastolic blood pressure >90 mm Hg

*** Including headaches, cough, fever, suspected and confirmed malaria

**** Active STI, including candidiasis, one possible HSV-1 infection, lower abdominal pain in women, and Bartholin's abscess

‡ Including suspected and confirmed TB, pneumonia

† Includes 125/184 (67.9%) medical exclusions not linked to infectious disease (e.g., trauma, diabetes, cancer)

§ Includes 4/17 (23.5%) medical exclusions not linked to infectious disease (3 peripheral neuropathy and 1 inebriation at time of visit)

Among enrolled volunteers, the prevalence of HBsAg was 4.4% (106/2387) and of Hepatitis C antibody was 4.0% (95/2387), with significant variations across sites (Table 3). Dual Hepatitis B and C infections were uncommon (n = 4). Fifty-five volunteers (2.3%) were RPR positive, and this did not vary by gender. After 27 self-reported pregnant women were screened out prior to enrollment, an additional 1.6% (18/1140) enrolled women were excluded from analysis because they had positive urine pregnancy tests.

## Discussion

AIDS vaccine trials in Africa have not typically employed local laboratory reference intervals for screening of potential volunteers and evaluation of adverse events during follow up [personal communication, C Schmidt]. Several studies in the literature have suggested that individuals of African origin have different laboratory reference intervals for a few hematology parameters compared to Caucasians in industrialized countries. Africans have been described as having lower platelet counts [6], [15], [16], lower neutrophil counts [5], [17]–[19] and lower CD4 T-cell counts [4], [20]. Differences in African and Caucasian populations have also been described for biochemistry, including those for uric acid [21], total protein [22], globulins and calcium [23]. Much of this research is older, and reference interval intervals may differ due to differences between laboratories (e.g., different test methods), study design (e.g., sampling conditions, criteria for selection of individuals) and geographical areas (e.g., differences in temperature, altitude and endemic diseases). Prior to the work summarized in this paper, few published studies followed recommended guidelines for the establishment of reference intervals as suggested by working groups such as the CLSI. In our study, careful consideration was given to standardization of analytical methods and instrumentation. Our study is the first large scale, multi-site study conducted to the principles of GCLP to characterize local laboratory reference intervals applicable to potential volunteers in African clinical trials.

Nearly one-third (883/2990, 29.5%) of all persons screened for the current study were not eligible for analysis. Differences in the proportion of participants found ineligible both before and after screening were observed between gender, between countries, within country and between rural and urban areas (Table 2). Infectious diseases accounted for more than half of all potential volunteers who were not eligible for analysis (513/883, 58%), including Hepatitis B and C (23%), splenomegaly (11%), possible malaria (6%, data on malaria diagnosis was not collected), STI (4%) and respiratory tract infections (4%). The high rate of splenomegaly in the Masaka region of Uganda may suggest underlying infections, and further investigation is underway. In many African regions, the combination of parasitic infections such as schistosomiasis and malaria is responsible for high rates of splenomegaly; this may help to explain our observed geographical differences [24], [25]. However, hematological disorders and idiopathic splenomegaly have also been reported [26]. Hepatitis C antibody prevalence was unexpectedly high in 2 sites, Masaka (9%) and Kilifi (8%), and Hepatitis B antigen prevalence was also high in Kilifi (10%). No confirmatory tests of hepatitis B or C infections were conducted, and since false positive results have been reported [27], [28] our results require further evaluation. Another common exclusion factor was the presence of hypertension. This is consistent with other studies in African populations where high rates of essential hypertension have been documented [29], [30].

A goal of the study was to include equivalent numbers of men and women. More women were screened out, and more men were excluded from analysis, leaving a final cohort with a balanced gender makeup (48% women). The higher rate of female screen-outs was at least in part due to the 27 women who screened out due to reported pregnancy and the 44 who did not return when rescheduled due to menstruation at screening. Relatively few women (18) tested positive for pregnancy once enrolled, while the prevalence of Hepatitis B & C was significantly higher among enrolled men than women.

The selection of study populations varied by site, and potential volunteers were frequently selected from participants of ongoing research, or individuals with a previous interest in participating in research studies. Therefore, the selection of volunteers for this study does not represent a random sample of the local residents. The exception to this is Masaka, where study volunteers were recruited from the general population after the entire adult population of three rural villages was informed of upcoming research activities. The MRC has been working in these communities for some time, and the many of the residents there have also participated in previous research activities [31]. The purpose of this study was to characterize laboratory values in individuals who might otherwise have participated in vaccine clinical trials, and this selection bias may therefore limit the generalizability of these results to the general populations of each locale.

Data were collected on rainy versus dry season, with some sites participated in a sub-study that included repeating laboratory tests in both the rainy and dry seasons. These data will be presented in a future report. It should be noted that the sites differ significantly in altitude above sea level, ranging from sea level to 1680 meters (Figure 1). This difference, as well as others such as demographics and season will be taken into consideration for future analyses comparing reference intervals across sites.

Several laboratory implementation lessons were learned during the course of this study. To assure data were comparable across sites, significant training of staff in the principles of GCLP and laboratory management was conducted. Cross validation studies are essential prior to implementation of study testing to ensure that all sample processing and analytical procedures are conducted appropriately. Our work highlighted several problems of both a pre-analytical and analytical nature. We found that reagent supply and instrument maintenance were problematic at some sites and finding proper technical support and in country procurement sources could be difficult. To maximize cost-effectiveness and practicality in some cases, we allowed local product availability to dictate reagent use in some cases in lieu of universal standardization (Table 1). EQA programs helped maintain satisfactory analytical performance in real-time, so site visits from the EQA laboratories are essential to allow for troubleshooting and additional training.[Fn fn1]

## Footnotes

This work was previously presented as a preliminary report at the AIDS Vaccine Conference 2006, Amsterdam, The Netherlands (Poster P13e03) and as part of a Satellite Session prior to the AIDS Vaccine Conference 2007, Seattle, WA, USA. This manuscript has not been published in whole or in part elsewhere.
